# First-Line Enfortumab Vedotin Plus Pembrolizumab in Platinum-Ineligible Urothelial Carcinoma: A Three-Case Series

**DOI:** 10.7759/cureus.104355

**Published:** 2026-02-27

**Authors:** Daisuke Igarashi, Go Kaneko, Kimiharu Takamatsu, Suguru Shirotake, Masafumi Oyama

**Affiliations:** 1 Uro-Oncology, Saitama Medical University International Medical Center, Hidaka, JPN

**Keywords:** enfortumab vedotin, maintenance hemodialysis, pembrolizumab, platinum-ineligible, renal impairment, urothelial carcinoma

## Abstract

Locally advanced or metastatic urothelial carcinoma (la/mUC) has a poor prognosis. In routine clinical practice, platinum-based chemotherapy has generally been used as first-line treatment. Enfortumab vedotin plus pembrolizumab (EVP) has recently been introduced as another first-line option; however, patients who are platinum-ineligible, such as those with severe renal dysfunction and/or poor performance status, are often underrepresented in clinical studies, and the optimal systemic therapy for this group remains uncertain. Herein, we report three platinum-ineligible patients with la/mUC who received standard-dose EVP as first-line therapy. Platinum ineligibility was defined as creatinine clearance (CrCl) < 30 mL/min and/or Eastern Cooperative Oncology Group performance status (ECOG PS) ≥ 3. In Case 1 (hemodialysis; ECOG PS 1), EVP resulted in improvement of bone metastases and durable disease control, with manageable adverse events (AEs), including grade 2 skin toxicity, dysgeusia, hypothyroidism, and transient peripheral sensory neuropathy that resolved after temporary treatment interruption. In Case 2 (CrCl 23.1 mL/min; ECOG PS 2), EVP induced a partial response in the bladder and nodal lesions; however, treatment was complicated by early-onset grade 4 immune-related nephritis requiring high-dose corticosteroids. Best supportive care was adopted at progression. In Case 3 (ECOG PS 3), rapidly progressive renal pelvic la/mUC showed early disease progression with spinal cord compression despite EVP, leading to the patient being transitioned to best supportive care. Across all three cases, no life-threatening toxicity was clearly attributable to enfortumab vedotin. These observations suggest that standard-dose EVP may be a feasible first-line option for carefully selected platinum-ineligible patients, when administered with close monitoring, flexible dose adjustment, and prompt management of immune-related and other AEs.

## Introduction

Locally advanced or metastatic urothelial carcinoma (la/mUC) is a lethal disease, with more than half a million new bladder cancer cases and over 200,000 deaths worldwide each year [1]. Most patients ultimately require systemic therapy, and long-term survival is poor.

For several decades, the standard systemic treatment for advanced UC has been platinum-based chemotherapy. The recent JAVELIN Bladder 100 trial showed that, among patients who had no disease progression after 4-6 cycles of platinum-based chemotherapy, maintenance avelumab significantly prolonged overall survival (OS) compared with best supportive care alone; however, the magnitude of this survival benefit was modest [2]. Furthermore, this strategy is not applicable to patients who are considered platinum-ineligible, as they were excluded from the trial.

More recently, the phase III EV-302/KEYNOTE-A39 trial demonstrated that enfortumab vedotin plus pembrolizumab (EVP) achieves significantly superior OS and response rates than platinum-based chemotherapy [3]. EVP is now endorsed as a preferred first-line regimen in major international guidelines [4,5]. However, patients considered truly ineligible for platinum-based chemotherapy, such as those with a creatinine clearance (CrCl) < 30 mL/min and/or an Eastern Cooperative Oncology Group performance status (ECOG PS) ≥ 3, were excluded from this trial. Therefore, optimal systemic therapy for this high-risk population remains an important unmet medical need. Moreover, data specifically addressing the efficacy and safety of EVP in platinum-ineligible patients are needed. Against this background, we report a case series of three platinum-ineligible patients with la/mUC who received first-line EVP.

## Case presentation

Case 1

A 59-year-old man with a history of childhood surgery for ileus was referred to our hospital for systemic therapy. His ECOG PS at referral was 1. Four years earlier, at another institution, he had been diagnosed with muscle-invasive bladder cancer and left renal pelvic cancer. He received neoadjuvant chemotherapy with gemcitabine plus carboplatin, followed by radical cystectomy, left nephroureterectomy, and right cutaneous ureterostomy. Histopathological examination showed high-grade UC of the bladder, ypT1N0, and high-grade UC of the left renal pelvis, ypTis. After surgery, his renal function gradually declined, and maintenance hemodialysis was introduced three years before presentation.

**Figure 1:**
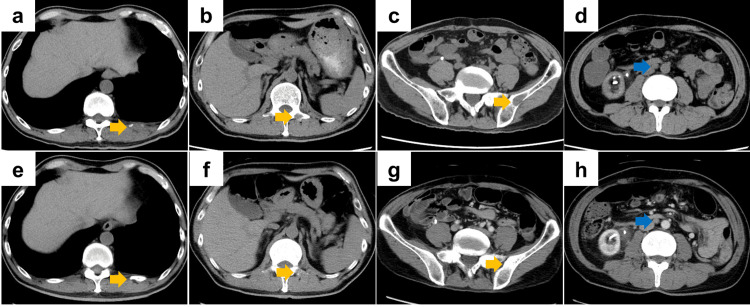
Computed tomography (CT) findings in Case 1 (a-d) Baseline contrast-enhanced CT at the time of diagnosis of metastatic disease. (a) Lytic lesion in the right 11th rib, (b) osteolytic lesion in the second lumbar vertebra, (c) osteolytic lesion in the left iliac bone, and (d) para-aortic lymph node metastasis. (e-h) CT after 4.2 months of first-line enfortumab vedotin plus pembrolizumab. The metastatic lesions in the 11th rib and left iliac bone are reduced in size, whereas the second lumbar vertebral lesion and para-aortic lymph node metastasis remain radiologically stable. Yellow arrows indicate bone metastases. The blue arrow indicates a para-aortic lymph node metastasis.

During follow-up, computed tomography (CT) revealed multiple bone metastases and para-aortic lymph node metastases. The patient was referred to our institution for systemic treatment. Restaging contrast-enhanced CT demonstrated bone metastases in the 11th rib, second lumbar vertebra, and left iliac bone, as well as enlarged para-aortic lymph nodes (Figures 1a-1d).

We initiated EVP at standard doses, with a treatment plan to perform hemodialysis approximately 24 hours after each EVP administration. During treatment, the patient developed several adverse events (AEs), including Common Terminology Criteria for Adverse Events (CTCAE) grade 1 fatigue and grade 2 skin toxicity, dysgeusia, and hypothyroidism. Contrast-enhanced CT performed 4.2 months after starting EVP showed no obvious change in the second lumbar vertebral metastasis or in the para-aortic lymph node metastases, whereas the lesions in the 11th rib and left iliac bone had decreased in size, indicating partial improvement of the osseous disease (Figures 1e-1h). At approximately the same time, grade 2 peripheral sensory neuropathy developed. Therefore, both drugs were temporarily withheld 4.7 months after EVP initiation. No specific pharmacologic treatment was given for neuropathy; however, the symptoms improved spontaneously to grade 1 within 1.7 months of treatment interruption, and EVP was restarted without dose reduction of EV.

After re-initiation, EVP has been continued for approximately four months. No new AEs have occurred; there has been no worsening of peripheral sensory neuropathy; and the disease, including bone lesions and para-aortic lymph node involvement, has remained well controlled.

Case 2

A 74-year-old man with a history of hypertension and type 2 diabetes mellitus had previously undergone percutaneous coronary intervention for myocardial infarction and implantation of a cardioverter-defibrillator. He underwent laparoscopic left nephroureterectomy for left distal ureteral cancer. Histopathological examination revealed invasive high-grade UC, pT3Nx, with lymphovascular invasion and negative surgical margins.

At 11.5 months after surgery, intravesical recurrence was detected, and transurethral resection of the bladder tumor (TURBT) was performed. The pathological diagnosis was UC, pT1, with lymphovascular invasion. As adjuvant therapy, intravesical Bacillus Calmette-Guérin instillation (Immunobladder® 80 mg, once weekly for six doses) was administered.

**Figure 2:**
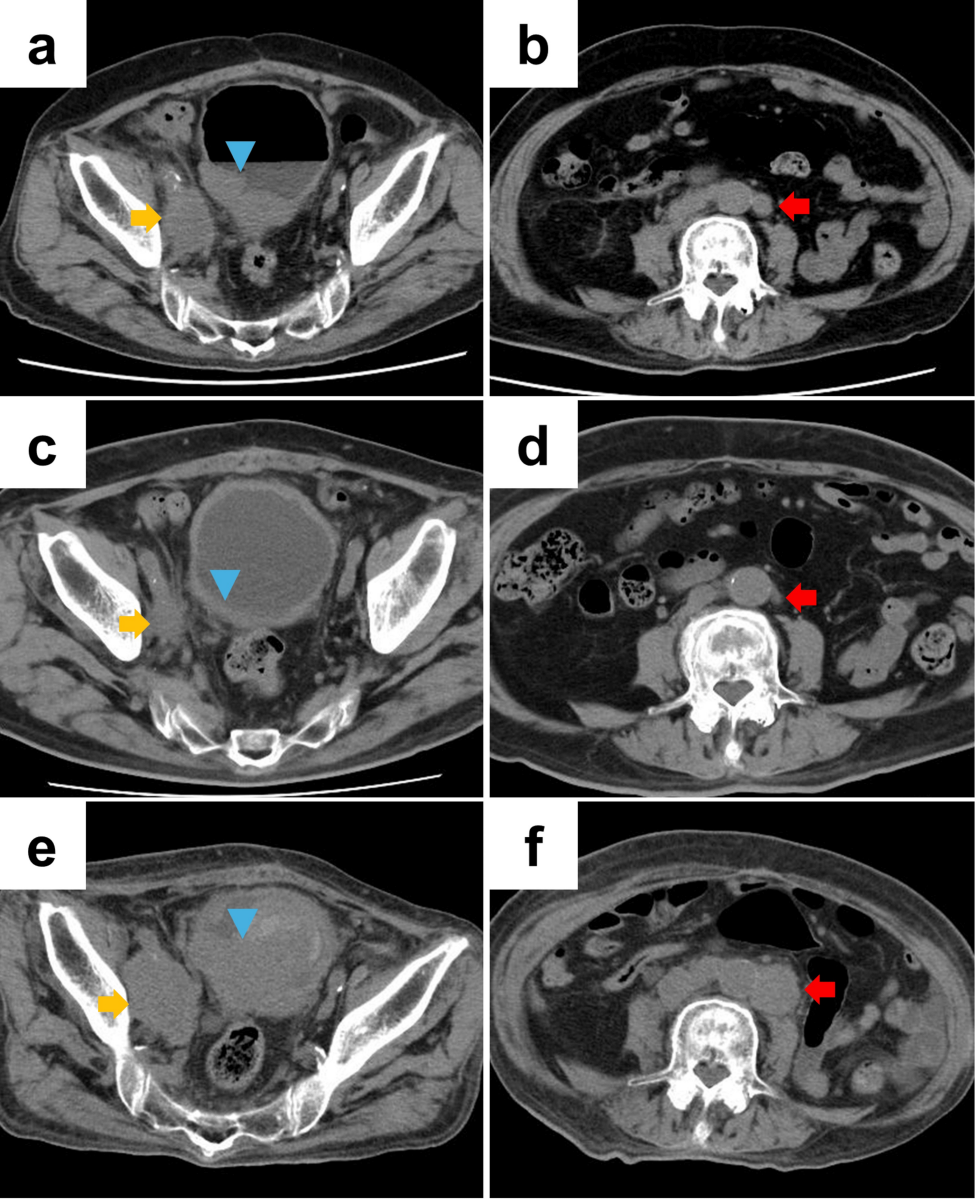
Computed tomography (CT) findings in Case 2 (a, b) Baseline contrast-enhanced CT at the time of diagnosis of metastatic disease. (a) Bladder tumor with suspected extravesical extension and right obturator lymph node metastasis. (b) Para-aortic lymph node metastasis. (c, d) CT after two cycles of first-line enfortumab vedotin plus pembrolizumab (EVP) showing reduction in the size of the bladder tumor, right obturator lymph node metastasis, and para-aortic lymph node metastasis. (e, f) CT at 5.2 months after initiation of EVP, demonstrating marked regrowth of the bladder tumor and both nodal metastases, consistent with radiologic disease progression. The blue arrowhead, yellow arrow, and red arrow indicate the bladder tumor, right obturator lymph node metastasis, and para-aortic lymph node metastasis, respectively.

Follow-up contrast-enhanced CT performed 9.6 months after TURBT showed recurrent bladder tumor and metastases in the right obturator and para-aortic lymph nodes (Figures 2a, 2b). Systemic therapy was considered; however, the patient subsequently developed heart failure and pneumonia, requiring hospitalization and treatment at another hospital. After his general condition improved, EVP at standard doses was started 2.5 months after the diagnosis of metastases. At the start of EVP therapy, his ECOG PS was 2 and CrCl was 23.1 mL/min. During treatment, he developed CTCAE grade 2 skin toxicity and dysgeusia and grade 1 peripheral sensory neuropathy, which were managed with conservative treatment. After two cycles, contrast-enhanced CT demonstrated shrinkage of the bladder lesion and multiple lymph node metastases, and the response was assessed as a partial response according to the RECIST criteria (Figures 2c, 2d).

At the scheduled visit for the fourth cycle, 2.8 months after starting EVP, blood tests revealed CTCAE grade 4 renal impairment (CrCl 7.36 mL/min). Immune-related (ir) nephritis was suspected, and oral prednisolone at 1 mg/kg/day was initiated, resulting in gradual improvement in renal function.

During tapering of prednisolone, the patient was admitted 5.2 months after EVP initiation due to loss of appetite. Contrast-enhanced CT at admission showed marked enlargement of the bladder lesion and multiple lymph node metastases, consistent with radiologic disease progression (Figures 2e, 2f), accompanied by a decline in ECOG PS to 3. Further systemic chemotherapy was deemed inappropriate. The treatment strategy was changed to best supportive care, and he was transferred to another hospital.

Case 3

**Figure 3:**
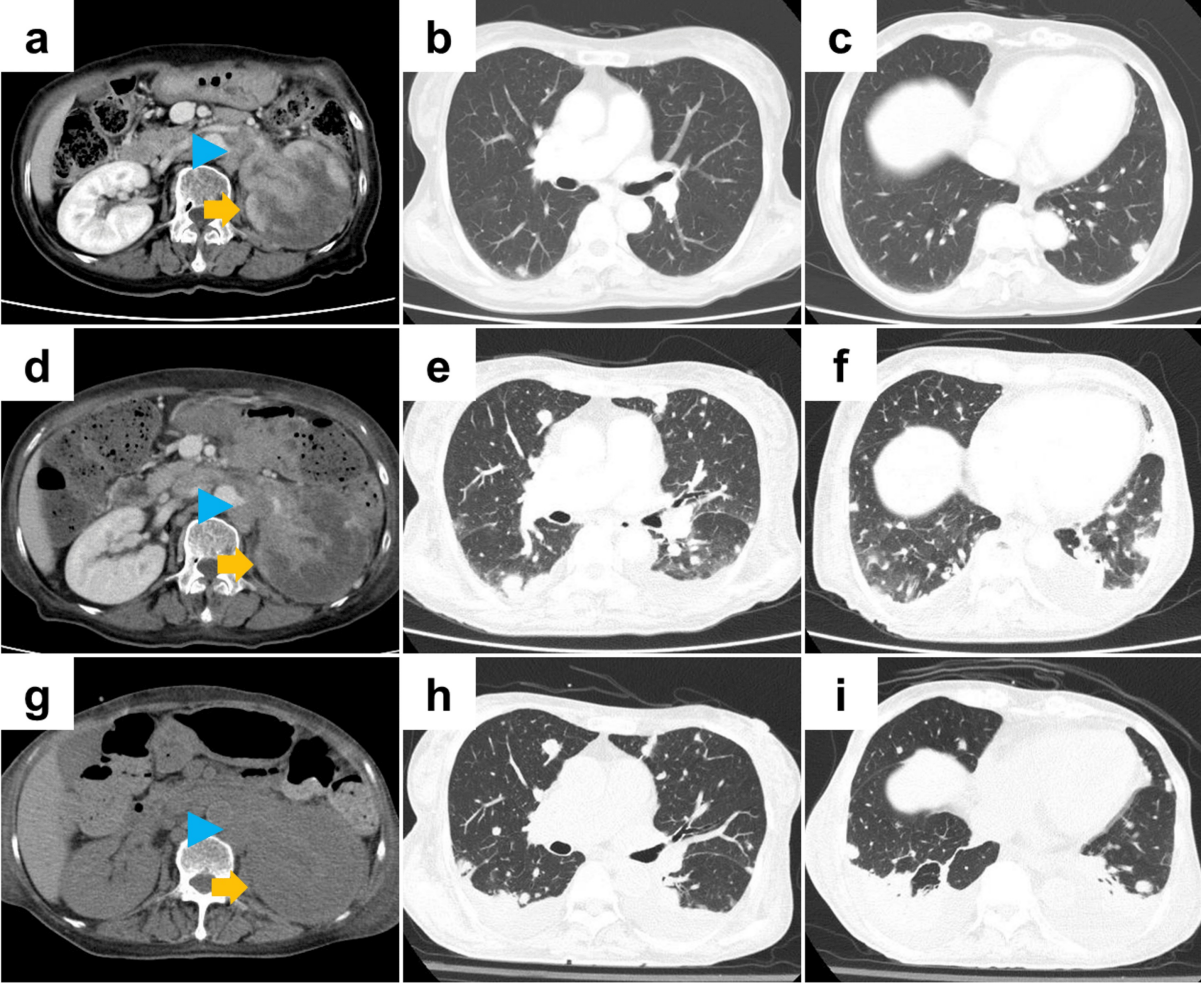
Computed tomography (CT) findings in Case 3 (a-c) Baseline contrast-enhanced CT at the time of diagnosis of metastatic disease. (a) Left renal pelvic mass infiltrating the renal parenchyma with suspected perirenal fat invasion, accompanied by multiple renal hilar lymph node metastases. (b, c) Multiple bilateral pulmonary metastases. (d-f) CT performed 8 days after renal biopsy. (d) Interval enlargement of renal hilar lymph node metastases. (e, f) Increase in both the number and size of multiple lung metastases, with new bilateral pleural effusions. (g-i) CT obtained 16 days after initiation of first-line enfortumab vedotin plus pembrolizumab, showing progressive disease. (g) Further enlargement of the left renal pelvic primary tumor and renal hilar lymph node metastases. (h, i) Continued increase in pulmonary metastases and worsening bilateral pleural effusions. Yellow arrows indicate the left renal pelvic primary tumor. Blue arrowheads indicate renal hilar lymph node metastases.

A 75-year-old woman with a history of type 2 diabetes mellitus and hypothyroidism presented to a local clinic with loss of appetite and fatigue. CT revealed a left renal mass and multiple lung nodules, and she was referred to our hospital for further evaluation and treatment. Urine cytology suggested high-grade UC according to the Paris system. Contrast-enhanced CT demonstrated an enhancing left renal pelvic mass infiltrating the renal parenchyma and perirenal fat, and multiple renal hilar lymph node and lung metastases (clinical stage, cT4N2M1) (Figures 3a-3c). Ultrasound-guided biopsy of the renal mass was performed, which confirmed renal pelvic UC.

Eight days after the biopsy, she developed worsening left flank and back pain, necessitating emergency admission. Contrast-enhanced CT showed enlargement of the renal hilar lymph nodes, as well as an increase in the number and size of pulmonary metastases, suggesting rapid disease progression (Figures 3d-3f). Opioid analgesics were introduced for pain control. Her ECOG PS was 3. She was considered ineligible for platinum-based chemotherapy.

First-line EVP therapy at standard doses was initiated. No clear acute treatment-related AEs were observed during the limited observation period immediately after EVP initiation; however, 16 days after starting EVP, she developed weakness of the lower limbs. CT revealed a new metastasis in the 12th thoracic vertebra with epidural extension, as well as further progression of the primary tumor, renal hilar lymph node metastases, and lung metastases (Figures 3g-3i). Overall, disease progression was diagnosed. Palliative radiotherapy was administered to the vertebral lesion, but lower limb paralysis progressed, and her activities of daily living declined markedly. Intensive systemic therapy was no longer considered feasible, and the treatment strategy was changed to best supportive care. She was transferred to another hospital 1.3 months after initiation of EVP therapy.

## Discussion

This case series describes three patients with la/mUC who were considered platinum-ineligible and received standard-dose EVP as first-line therapy. Across all three cases, no severe AEs were clearly attributable to EV itself, suggesting that, with appropriate supportive care and dose modification, standard-dose EVP can be initiated even in this high-risk population. Case 2 developed early-onset, severe ir nephritis, underscoring that close monitoring for irAEs is mandatory from the start of treatment. Taken together, these cases provide real-world support that, with rigorous toxicity management and careful patient selection, EVP may be a feasible first-line option for selected platinum-ineligible patients.

Recent real-world evidence underscores the particularly vulnerable status of patients ineligible for platinum-based therapy. In an electronic health record-based cohort of 4,270 la/mUC patients, Gupta et al. identified 477 (11%) who met the strict criteria for platinum ineligibility (ECOG PS ≥ 3 and/or severe renal dysfunction); in this subgroup, median OS and progression-free survival (PFS) were only 5.1 and 3.4 months, consistently worse than in platinum-eligible patients regardless of whether the first-line therapy was chemotherapy or PD-1/PD-L1 monotherapy [6]. Similarly, Pond et al. reported an objective response rate of 27.9% and a median OS of 45 weeks among 79 platinum-ineligible patients treated with first-line PD-1/PD-L1 inhibitor monotherapy [7]. Together, these findings suggest that platinum-ineligible la/mUC patients are a biologically and clinically fragile population with poor outcomes, in whom current immune checkpoint inhibitor (ICI) monotherapy confers only limited benefit and more effective, better-tolerated first-line regimens are urgently needed.

Because patients with CrCl < 30 mL/min or poor PS were excluded from EV-302, robust trial data on the efficacy and safety of EVP in truly platinum-ineligible patients are lacking. Real-world evidence is now beginning to emerge. In an electronic health record-based cohort of 462 patients treated with first-line EVP, 65 patients (14.1%) had baseline CrCl < 30 mL/min. In this subgroup, OS, PFS, and treatment interruption-free survival were not significantly inferior to those observed in patients with CrCl ≥ 30 mL/min, suggesting that EVP retains efficacy in the setting of advanced renal dysfunction [8]. In addition, a recent case report described a patient with mUC on chronic hemodialysis who received standard-dose EVP on non-dialysis days and experienced only grade 2 pruritus and grade 1 fatigue, with no unexpected safety signals [9]. These findings, together with our three cases, support EVP as a promising first-line option for carefully selected patients who cannot receive platinum-based chemotherapy. Prospective studies specifically designed for this high-risk population are needed to establish its risk-benefit profile more definitively.

Real-world data also indicate that, in the later-line setting for la/mUC, pembrolizumab monotherapy and EV monotherapy can be administered even in patients with markedly impaired renal function, with efficacy and safety that are not necessarily inferior to those observed in patients without severe renal dysfunction [10-12]. Furthermore, reports that include patients with poor ECOG PS suggest that, with careful patient selection and close follow-up, these regimens may provide meaningful clinical benefit [11,13]. At our institution, we have treated patients with severe renal impairment, including those on maintenance hemodialysis, and patients with ECOG PS 3 using pembrolizumab or EV monotherapy. In contrast, because patients with poor PS and/or severe renal dysfunction would have been excluded from the EV-302/KEYNOTE-A39 trial and may be at increased risk of serious AEs, we do not routinely recommend EVP in this population. EVP is considered only exceptionally, after thorough shared decision-making with the patient and family (including best supportive care), when active treatment is strongly desired despite the risks and strict monitoring is feasible. Case 3 was such an exception: rapid disease progression and worsening pain from the enlarging primary tumor led to deterioration of ECOG PS to 3 before treatment initiation. After discussion of both EVP and best supportive care, EVP was initiated under close monitoring. In particular, during the early treatment phase, we carefully monitor for skin toxicity, peripheral neuropathy, and early irAEs, and we reduce the EV dose or temporarily interrupt one or both agents as needed to prioritize safety while maintaining treatment continuity and efficacy.

The optimal systemic therapy for platinum-ineligible la/mUC remains to be elucidated. Prospective registries and clinical trials that actively enroll platinum-ineligible patients are needed to assess the efficacy and safety of EVP in this setting. Future studies should not only evaluate antitumor efficacy but also place greater emphasis on patient-reported outcomes and treatment burden, given that many of these patients are elderly, frail, and highly vulnerable to toxicity. Until such evidence is available, treatment decisions must be individualized, taking into account frailty, comorbidities, organ function, symptom burden, and patient preferences, with explicit discussion of best supportive care when appropriate. Based on our experience, EVP may be considered only in highly selected platinum-ineligible patients under strict monitoring, with flexible dose adjustment and proactive AE management. However, this case series should not be interpreted as evidence supporting routine EVP use or indication expansion in patients outside standard trial-eligible populations; rather, Case 3 is presented as an exceptional and cautionary case that highlights the difficulty of treatment selection and the need for extreme caution in highly vulnerable patients.

## Conclusions

Standard-dose EVP may represent a viable first-line treatment option for carefully selected platinum-ineligible patients with la/mUC when administered under rigorous toxicity surveillance. In addition to EV-related AEs, serious and potentially life-threatening irAEs can occur early after treatment initiation; thus, meticulous monitoring and prompt intervention are essential. Until prospective evidence is available, individualized care grounded in proactive supportive measures, flexible dose modification, and shared decision-making is critical to maximize clinical benefit while minimizing harm in this highly vulnerable group.
